# Intergenerational Relationships, Social Support, and Psychological Well-Being Among Korean Older Adults

**DOI:** 10.1093/geroni/igaa057.1608

**Published:** 2020-12-16

**Authors:** Yooumi Lee, Janet Wilmoth

**Affiliations:** Syracuse University, Syracuse, New York, United States

## Abstract

This study investigates whether intergenerational relationships and social support improve the psychological well-being of Korean older adults. We examine whether intergenerational relationships and social support directly influence psychological well-being and the extent to which they mediate the distressing consequences of life events such as declining health and recent widowhood. Using longitudinal data from the 2006 to 2016 Korean Longitudinal Study of Aging, we explore depression trajectories among individuals who are 60 or older with at least one living adult child at baseline. Specifically, we converted data from 5,383 older adults into a person-period file with 24,726 observations over a ten-year period. Then we estimated linear growth curve models of depression trajectories separately for men and women using the Center for Epidemiologic Studies Depression Scale (CES-D). Results from the hierarchical linear models indicate that declining health and recent widowhood are positively related to depressive symptoms. Satisfactory intergenerational relationships and social support in the form of personal interactions and proximate living arrangements with adult children decrease depressive symptoms of older parents, especially among women. We conclude that the psychological benefits of intergenerational relationships and social support are contingent upon the vulnerability of older adults and discuss the implications for public policy.

